# Effects of Functional Magnetic Stimulation on Pain, Function, and MRI-Derived Outcomes in Athletes with Tibial Bone Stress Injury: A Randomized Controlled Trial

**DOI:** 10.3390/jfmk11030317

**Published:** 2026-08-14

**Authors:** Dimitrios Lytras, Ioannis Algiounidis, Vasileios Georgoulas, Konstantinos Kasimis, Georgia Maria Kamparoudi, Georgios Tsigaras, Georgia Vergidou, Nikolaos Sidiropoulos, Georgia Tarfali, Ilias Kallistratos, Paris Iakovidis

**Affiliations:** 1 Laboratory of Biomechanics & Ergonomics, Department of Physiotherapy, Faculty of Health Sciences, International Hellenic University, Alexander Campus, 57400 Sindos, Thessaloniki, Greece; vasilisgeorgoulas@gmail.com (V.G.); kkasimis@ihu.gr (K.K.); piakov@ihu.gr (P.I.); 2 Giannitsa General Hospital, 58100 Giannitsa, Pella, Greece; giannisalgiounid@gmail.com; 3 Department of Physiotherapy, Faculty of Health Sciences, International Hellenic University, 57400 Sindos, Thessaloniki, Greece; gkamparoudi@ihu.gr (G.M.K.); vergidou.myphysio@gmail.com (G.V.); nikos.sid@gmail.com (N.S.); 4 Laboratory of Basic and Applied Research in Physiotherapeutic Rehabilitation, Department of Physiotherapy, Faculty of Health Sciences, International Hellenic University, 57400 Sindos, Thessaloniki, Greece; tsigarasg@ihu.gr (G.T.); elikall@ihu.gr (I.K.); 5 School of Health Sciences, Queen Margaret University, Edinburgh EH21 6UU, UK; gtarfali@gmail.com

**Keywords:** bone stress injury, tibial bone marrow edema, functional magnetic stimulation, magnetic resonance imaging, rehabilitation, athletes

## Abstract

**Background:** Tibial bone stress injury (TBSI) with associated bone marrow edema (BME) is a common and clinically challenging condition in athletes, often requiring prolonged load restriction and delayed return to sport. Evidence for adjunctive interventions that improve both symptoms and MRI-derived recovery remains limited. The aim of this study was to investigate whether adding Functional Magnetic Stimulation (FMS) to standardized rehabilitation improves pain, function, and MRI-derived outcomes over 16 weeks in athletes with MRI-confirmed TBSI. **Materials and Methods:** Forty athletes with Fredericson grade 2–3 TBSI were randomized to FMS plus rehabilitation (*n* = 20) or rehabilitation alone (*n* = 20). Both groups completed a 4-week load-based rehabilitation program, while the FMS group additionally received eight 30 min FMS sessions at 40 Hz. Outcomes were assessed at baseline, 4 weeks, and 16 weeks. The primary outcome was activity-related pain (NPRS), while secondary outcomes included lower-limb function (LEFS-GR), BME extent, and Fredericson grade. Continuous outcomes were analyzed using two-way mixed ANOVA, whereas Fredericson grade was analyzed using an ordinal generalized estimating equation model. The level of statistical significance was set at *p* < 0.05. **Results:** Groups were comparable at baseline. Significant group × time interactions favored FMS for NPRS, F(2, 76) = 12.46, *p* < 0.001, η^2^p = 0.247; LEFS-GR, F(1.08, 41.09) = 81.56, *p* < 0.001, η^2^p = 0.682; and BME extent, F(1.44, 54.85) = 49.33, *p* < 0.001, η^2^p = 0.565. At 16 weeks, the FMS group showed lower NPRS scores, higher LEFS-GR scores, and lower BME extent than controls. Fredericson grade also showed a significant group × time effect, Wald χ^2^(2) = 20.38, *p* < 0.001. The FMS group had significantly lower cumulative odds of classification in a higher Fredericson grade at 4 weeks (OR = 0.045, 95% CI: 0.012–0.163, *p* < 0.001), whereas the corresponding difference at 16 weeks remained directionally favorable but did not reach statistical significance (OR = 0.033, 95% CI: 0.001–1.029, *p* = 0.052). **Conclusions:** The addition of FMS to load-based rehabilitation was associated with greater clinical and functional improvements, greater reductions in BME extent, and a more favorable longitudinal trajectory in MRI severity classification than rehabilitation alone in athletes with TBSI. However, because the study did not include a sham-FMS condition and participants could not be blinded, the self-reported pain and functional outcomes should be interpreted cautiously. FMS may represent a useful adjunct to structured rehabilitation, although sham-controlled and longer-term studies should determine its effects on return-to-sport progression and recurrence risk.

## 1. Introduction

Tibial bone stress injury (TBSI) is a common overuse-related bone injury in athletic populations, particularly in running and other repetitive-impact sports [1,2]. The tibia is among the most frequently affected skeletal sites, accounting for approximately half of lower-limb bone stress injuries in athletes and physically active individuals [1,2,3]. TBSI develops when repetitive submaximal mechanical loading exceeds the capacity for physiological bone remodeling and repair, leading to microdamage accumulation along a pathological continuum from early stress reaction to overt stress fracture [1,3,4]. Clinically, these injuries typically present with localized loading-related pain, reduced training tolerance, functional limitation, and potentially prolonged absence from sport [1,4,5]. Bone stress injury reflects an imbalance between load-induced microdamage and remodeling-mediated repair, in which bone resorption may temporarily exceed bone formation [5,6,7]. Because exercise-related tibial pain has historically been described using overlapping terms, bone marrow edema should be understood primarily as an MRI finding rather than a standalone diagnosis [5,7], while contemporary literature increasingly supports the umbrella term bone stress injury to improve diagnostic consistency [3,6].

A major challenge in the evaluation of exercise-related tibial pain is differentiating clinically meaningful tibial bone stress injury from other causes of lower-leg pain, particularly medial tibial stress syndrome. Although patient history and physical examination remain essential, clinical findings alone may be insufficient to confidently determine the presence, severity, or anatomical extent of underlying bone injury, especially in early-stage presentations or in athletes with overlapping symptoms [3,5,8]. Accordingly, magnetic resonance imaging (MRI) is regarded as the modality of choice for confirming and staging bone stress injury, as it allows for early detection of periosteal and bone marrow signal changes, assessment of injury severity, and classification according to MRI-based grading systems such as the Fredericson classification, including early-stage lesions without a visible cortical fracture line [3,5,7].

Management of TBSI is predominantly conservative and is centered on activity modification, relative unloading, graded reloading, therapeutic exercise, identification and management of modifiable contributing factors, and progressive return to sport [6,9,10]. Within this framework, physiotherapy plays a central role, as it aims not only to reduce pain and restore function, but also to safely reintroduce the athlete to progressively increasing loads through controlled loading, functional retraining, strengthening, functional progression, and individualized return-to-running planning [6,9]. However, despite broad acceptance of conservative care, recovery and return-to-sport timelines remain variable and partly dependent on injury location and severity, and evidence remains limited regarding strategies that may safely optimize or accelerate clinical, functional, and return-to-sport recovery in athletes with TBSI [9,11].

In recent years, there has been growing interest in non-invasive adjunctive biophysical interventions that may complement standard load-based rehabilitation in bone stress injuries and other conditions associated with bone marrow edema. Among these, focused extracorporeal shock wave therapy (ESWT) has been explored in athletes and runners with bone stress injury, with promising but still limited evidence regarding symptom improvement and return to running or sport, primarily derived from case series, retrospective studies, and narrative or clinical reviews that consider it a potential adjunctive option [4,12]. In parallel, other forms of biophysical stimulation, such as pulsed electromagnetic fields (PEMFs), have been investigated in conditions associated with bone marrow edema, with reports of improvements in pain, function, and, in some cases, MRI findings; however, the available evidence has mainly been derived from small studies, heterogeneous clinical populations, and non-tibial anatomical sites, rather than specifically from athletes with tibial bone stress injury [13,14,15]. Therefore, despite the increasing interest in these biophysical interventions, the current evidence remains fragmented, underscoring the need for further investigation of targeted non-invasive approaches in well-defined athletic populations.

One emerging non-invasive approach in rehabilitation is Functional Magnetic Stimulation (FMS), a form of extracorporeal electromagnetic stimulation that delivers high-intensity pulsed magnetic fields capable of inducing electric currents in excitable neuromuscular tissues. Although its application has been explored primarily in other areas of rehabilitation and musculoskeletal medicine rather than in bone stress injuries specifically, FMS has been proposed as a modality that may favorably influence pain modulation, neuromuscular activation, functional performance, and rehabilitation tolerance through mechanisms related to electromagnetic induction and the bioelectrical responsiveness of excitable tissues [16,17]. Preliminary clinical evidence in individuals with lumbar disc herniation with radiculopathy has shown short-term improvements in pain, lumbar mobility, neural mechanosensitivity, and functional outcomes following FMS application [18]. In critically ill patients, a related approach, referred to in the original study as functional magnetic neuromuscular stimulation, was associated with peripheral neuromuscular activation and preservation of muscle thickness [17]. However, direct evidence regarding the use of FMS in athletes with tibial bone stress injury remains extremely limited.

Although FMS and PEMFs differ in stimulation intensity, treatment parameters, and primary physiological targets [13,14,15,16,17], the PEMF literature provides indirect biological and clinical plausibility for examining MRI-derived changes following electromagnetic stimulation in conditions associated with bone marrow edema [13,14,15]. However, because FMS primarily targets excitable neural and muscular tissues and produces neuromuscular activation [16,17], a direct effect on bone remodeling or edema resolution cannot be assumed. Any influence on MRI-derived recovery may instead occur indirectly through improved symptom control, greater loading tolerance, and more consistent participation in progressive rehabilitation. Accordingly, BME extent and Fredericson grade were included as exploratory secondary outcomes to determine whether clinical and functional improvement was accompanied by MRI-derived changes, rather than on the assumption that FMS directly accelerates bone healing.

Despite growing interest in non-invasive biophysical interventions, the available literature remains limited and heterogeneous regarding athletes with MRI-confirmed tibial bone stress injury. In particular, there is insufficient evidence on whether adding FMS to a structured rehabilitation program may improve clinical, functional, and imaging outcomes over a clinically relevant follow-up period. On this basis, the present study evaluated FMS as an adjunct intended to support participation in progressive rehabilitation, while any association with MRI-derived recovery was considered exploratory rather than evidence of a direct effect on bone healing. Therefore, the aim of the present randomized controlled trial was to investigate whether the addition of FMS to a standardized rehabilitation program results in greater improvement in activity-related pain as the primary clinical outcome, and in lower-limb function and MRI-derived indicators of recovery as secondary outcomes, over a 16-week follow-up period compared with rehabilitation alone. Activity-related pain assessed using the NPRS was designated as the sole confirmatory outcome, whereas lower-limb function assessed using the LEFS-GR, BME extent, and Fredericson grade were treated as exploratory secondary outcomes. The confirmatory hypothesis was that the FMS group would demonstrate greater improvement in activity-related pain over the 16-week follow-up than the control group, whereas the corresponding null hypothesis was that no statistically significant group × time difference would be observed for the NPRS. Directional improvements in the functional and MRI-derived outcomes were also anticipated, but these outcomes were interpreted as exploratory and were not assigned confirmatory inference.

## 2. Materials and Methods

### 2.1. Study Design

This study was conducted under the supervision of the Laboratory of Biomechanics & Ergonomics, Department of Physiotherapy, at the International Hellenic University, Thessaloniki, Greece, and was designed in accordance with the CONSORT statement for randomized clinical trials. The study followed a two-arm, parallel-group, randomized controlled trial design with blinded outcome assessment, three assessment time points, and a 1:1 allocation ratio between groups. The research protocol was approved by the Ethics Committee of the Department of Physiotherapy, Faculty of Health Sciences, International Hellenic University (approval number: EC-15/2025), and was prospectively registered at ClinicalTrials.gov (Identifier: NCT07234084). All participants provided written informed consent prior to enrollment. The completed CONSORT checklist is provided in the Supplementary Materials.

### 2.2. Recruitment, Randomization, and Blinding

Participants were recruited from outpatient sports medicine and physiotherapy settings in Thessaloniki, Greece. All potential participants were recreationally or competitively active athletes who had undergone prior orthopedic evaluation and MRI examination due to exercise-related tibial pain. Screening for study eligibility was performed by an orthopedic physician experienced in sports musculoskeletal injuries, based on clinical presentation and MRI findings consistent with TBSI. Before recruitment began, the allocation sequence was generated by an independent researcher using computer-generated permuted block randomization with randomly varying block sizes of four and six and a 1:1 allocation ratio. No stratification by sex, baseline Fredericson grade, or other participant characteristics was applied. The block sizes and allocation sequence were not disclosed to the personnel responsible for participant enrollment. Allocation concealment was ensured using sequentially numbered, opaque, sealed envelopes, which were prepared according to the allocation sequence. After eligibility was confirmed, written informed consent had been obtained, and the baseline assessment had been completed, each participant was assigned a unique study identification code. The next envelope in the sequence was then opened, and the participant was allocated to either the FMS plus rehabilitation group or the rehabilitation-only control group. Because of the nature of the interventions, blinding of the treating physiotherapists and participants was not feasible. However, clinical and functional outcomes were assessed by an independent assessor blinded to group allocation, whereas MRI outcomes were evaluated by an experienced orthopaedic surgeon who was also blinded to group allocation.

### 2.3. Participants

The inclusion criteria were: (1) age between 18 and 45 years; (2) participation in regular sports training at least three times per week during the previous six months; (3) MRI-confirmed TBSI classified as Fredericson grade 2 or grade 3 at baseline; (4) localized tibial pain provoked by loading activity; (5) pain intensity of at least 3/10 on the NPRS during activity-related loading; and (6) provision of written informed consent. The upper age limit of 45 years was selected pragmatically to restrict the sample to a relatively homogeneous population of actively training adults and to reduce heterogeneity related to age-associated differences in bone health, comorbidity burden, and recovery characteristics. This cutoff was a methodological eligibility criterion and was not intended to represent a discrete biological threshold. Bilateral tibial involvement was not specified as an exclusion criterion; however, no participant presented with clinically identified bilateral TBSI at enrollment. Consequently, all enrolled participants were evaluated and analyzed as unilateral cases, and the clinical and MRI-derived assessments referred to the affected tibia.

The exclusion criteria were: (1) Fredericson grade 4 injury or evidence of a visible fracture line on MRI; (2) BME attributed to infection, tumor, systemic inflammatory disease, or other non-mechanical causes; (3) history of fracture, surgery, or intra-articular/peri-osseous injection in the affected lower limb within the previous 12 weeks; (4) prior exposure to FMS or ESWT within the previous 8 weeks; (5) contraindications to magnetic stimulation, including pacemaker, implanted neurostimulator, cochlear implant, or significant metallic implant in or near the treatment area; (6) pregnancy or breastfeeding; and (7) any neurological, metabolic, cardiovascular, or systemic condition that could interfere with safe participation or outcome interpretation.

### 2.4. Measurements

All outcomes were assessed at baseline, 4 weeks, and 16 weeks by two assessors, both of whom were blinded to group allocation: one assessed the clinical and functional outcomes, whereas the other evaluated the MRI outcomes. Activity-related tibial pain during standardized treadmill walking was defined as the primary outcome. Lower-limb functional ability, MRI-derived BME extent, and Fredericson MRI grade were defined as secondary outcomes.

#### 2.4.1. Primary Outcome: Activity-Related Pain Assessment Using a Standardized Treadmill Walking Test

Activity-related tibial pain was assessed using an 11-point Numerical Pain Rating Scale (NPRS, 0–10), where 0 indicated “no pain” and 10 indicated “worst imaginable pain.” The assessment approach was conceptually based on previously published protocols in tibial stress-related and overuse conditions, in which pain has been quantified during standardized activity-based loading tasks, most commonly treadmill running, using numerical or visual analogue pain scales [19,20]. These protocols provide a methodological precedent for assessing symptom provocation under controlled mechanical loading, although the specific running-based tests used in MTSS research have not been fully validated as outcome measures and were not directly replicated in the present study.

Because the present study included athletes with active MRI-confirmed TBSI (Fredericson grades 2–3), and because early management of such lesions typically involves load restriction and avoidance of excessive impact loading, more provocative tests such as hopping or running were deliberately avoided at baseline for safety reasons. Therefore, the original concept of standardized treadmill loading with pain quantification was conservatively adapted to a low-impact walking protocol designed to minimize the risk of symptom exacerbation while still providing a controlled and standardized mechanical stimulus. No formal pilot testing, validation, or test–retest reliability assessment of this walking adaptation was conducted before initiation of the trial.

The standardized test consisted of 10 min of treadmill walking at 0% incline. At the baseline assessment, each participant selected a brisk but comfortable walking speed that could be maintained without undue discomfort. A self-selected rather than fixed or anthropometrically normalized walking speed was used to balance symptom provocation with participant safety in athletes with active MRI-confirmed TBSI. Because participants differed in habitual walking speed, body size, sport background, and symptom irritability, a single prescribed speed could have represented substantially different relative loading demands, potentially resulting in insufficient loading in some participants and excessive symptom provocation in others. The selected speed was therefore individualized at baseline to provide a brisk but tolerable stimulus and was kept identical for the same participant at the 4- and 16-week reassessments. This approach prioritized within-participant standardization across repeated assessments, although it did not eliminate between-participant variability in mechanical loading and symptom provocation. During the test, participants were instructed to focus specifically on pain arising from the affected tibia. The outcome of interest was peak tibial pain experienced during the walking task, recorded immediately after test completion or premature termination using the NPRS (0–10).

To ensure participant safety, the test was terminated prematurely if any of the following stopping criteria were met: (1) pain intensity reached or exceeded 4/10 on the NPRS; (2) the participant reported sharp, focal, or unusual pain; or (3) the participant requested to stop. This approach preserved the rationale of previously published activity-based pain assessments—namely, quantification of symptoms under standardized mechanical loading—while conservatively adapting the loading mode from running to walking to ensure clinical and ethical appropriateness in participants with active tibial bone stress injury [19,20].

#### 2.4.2. Secondary Outcomes: MRI Findings

MRI was used to assess lesion severity and MRI-derived structural changes over the 16-week follow-up period. All MRI examinations included T1-weighted and fluid-sensitive sequences (fat-suppressed T2-weighted or STIR), acquired using identical imaging parameters at all assessment time points. MRI outcomes included both a qualitative severity grading and a quantitative assessment of bone marrow edema (BME) extent.

TBSI severity was graded using the Fredericson classification system (grades 0–4), in which grade 0 indicates a normal examination, grade 1 indicates periosteal edema without bone marrow involvement, grade 2 indicates periosteal and bone marrow edema visible on fluid-sensitive sequences, grade 3 indicates more extensive periosteal and marrow edema visible on both T1-weighted and fluid-sensitive sequences, and grade 4 indicates the presence of a fracture line [5]. Only participants with grade 2 or 3 lesions at baseline were eligible for inclusion in the study. All MRI scans were evaluated by an experienced orthopaedic surgeon with expertise in musculoskeletal MRI, who was blinded to group allocation and assessment time point. For each examination, the highest (worst) Fredericson grade observed within the affected tibia was recorded and treated as an ordinal indicator of injury severity.

In addition to MRI grading, the extent of BME was quantified as a continuous imaging outcome, consistent with previous MRI-based bone stress injury studies in which MRI grade and BME size were recorded as radiological outcomes [21]. However, the specific single-slice maximum longitudinal measurement used in the present study was treated as an operational linear measure rather than as a formally validated surrogate of total edema volume. In the present study, BME extent was operationally defined as the maximum longitudinal extent (cm) of intramedullary edema within the tibia, measured on sagittal STIR or fat-suppressed T2-weighted images. For each MRI examination, the slice demonstrating the greatest visible longitudinal extent of intramedullary edema was identified. Using the digital measurement tool of the DICOM viewer, a linear measurement was obtained from the most proximal to the most distal point at which abnormal high signal intensity consistent with bone marrow edema was clearly visible. Only intramedullary edema was considered for this measurement; periosteal edema was not included. If no bone marrow edema was visible on the fluid-sensitive sequences, the BME extent was recorded as 0 cm.

All BME measurements were performed on calibrated DICOM images, in which spatial resolution is defined by pixel spacing parameters, allowing linear distances to be expressed in real-world units (mm/cm). A standardized measurement protocol and identical MRI acquisition parameters were applied across all time points, and the repeated-measures imaging analysis was based on repeated within-subject measurements across baseline, 4 weeks, and 16 weeks, thereby minimizing the potential influence of minor inter-scan variability.

To evaluate the reproducibility of the linear BME extent measurement, a post-hoc reliability analysis was conducted using MRI examinations from 30 randomly selected participants, with equal representation from the intervention and control groups. The MRI examinations of the same 30 participants at baseline, 4 weeks, and 16 weeks were reassessed. The original assessor repeated the BME extent measurements after a 5-day interval while blinded to the initial measurement values. A second independent assessor measured the same MRI examinations using the identical predefined measurement protocol and without access to the measurements of the original assessor.

Changes in Fredericson grade and BME extent across the three assessment time points were used as MRI-based outcome measures to describe the imaging response over the follow-up period.

#### 2.4.3. Secondary Outcome: Assessment of Lower-Limb Functional Ability

Lower-limb functional ability was assessed using the Greek version of the Lower Extremity Functional Scale (LEFS-GR), a patient-reported outcome measure designed to quantify functional limitations related to musculoskeletal disorders of the lower extremity [22,23]. The LEFS consists of 20 items assessing the degree of difficulty in performing everyday and basic sport-related activities, with each item scored on a 5-point Likert scale ranging from 0 (“extreme difficulty or unable to perform”) to 4 (“no difficulty”). The total score ranges from 0 to 80, with higher scores indicating better lower-limb functional ability [22].

The Greek version of the LEFS has been cross-culturally adapted and evaluated for reliability in Greek individuals with lower-limb musculoskeletal disorders, demonstrating excellent internal consistency (Cronbach’s α = 0.974) and excellent test–retest reliability (ICC = 0.986, *p* < 0.001) [23]. In the present study, the LEFS-GR was used to capture global lower-limb functional ability during recovery from TBSI. Although the LEFS is not a sport-specific performance measure and has not been specifically validated in athletes with TBSI, it provides a clinically relevant index of functional recovery at the level of everyday and basic functional activities and was therefore considered appropriate as a rehabilitation outcome. The questionnaire was administered at baseline, 4 weeks, and 16 weeks.

### 2.5. Experimental Protocols

#### 2.5.1. Physiotherapy Rehabilitation Program

Both groups followed the same standardized physiotherapy rehabilitation program over a period of four weeks, consisting of three supervised sessions per week (12 sessions in total). Each session lasted approximately 45 min and was delivered by licensed physiotherapists trained in the study protocol. The rehabilitation program was designed according to contemporary physiotherapy and sports rehabilitation principles for TBSI and was primarily informed by current published recommendations regarding graded loading, return-to-running criteria, symptom monitoring, and management of contributing factors [9]. Additional support for the symptom-guided loading rationale was drawn from broader evidence and clinical recommendations for exercise-related tibial overload conditions, particularly medial tibial stress syndrome [24].

The program followed a phased, symptom-guided progression across the 4-week intervention period. During the initial phase (week 1), emphasis was placed on relative unloading, symptom-guided activity modification, pain control, and restoration of pain-free basic lower-limb function. From the second week onward, participants progressed to a structured reloading phase, which included gradual increases in weight-bearing tolerance, lower-limb strengthening, mobility exercises, and controlled functional loading, provided that symptoms remained stable during and after treatment and no next-day exacerbation occurred. During the final phase of the program (weeks 3–4), loading demands were further progressed according to individual tolerance, with greater emphasis on functional retraining, improvement of load tolerance, and preparation for gradual return to higher-impact sport-related activity.

Across all phases, the rehabilitation program included: (1) relative unloading and symptom-guided activity modification, (2) progressive reloading of the affected lower limb, (3) therapeutic exercise targeting lower-limb strength, mobility, and load tolerance, and (4) individualized home-based exercise and return-to-activity instructions. Exercise progression followed a symptom-guided loading model, with progression permitted only when pain during and after loading remained within acceptable limits and no clinically relevant exacerbation was reported on the following day. Running, jumping, and other impact-based sport activities were temporarily modified during the intervention period and were reintroduced only as clinically tolerated within the structured progression. A detailed week-by-week overview of the rehabilitation program is provided in Supplementary Table S1.

After completion of the 4-week supervised rehabilitation program, no further standardized or investigator-supervised rehabilitation was provided before the 16-week reassessment. Participants received the same written guidance regarding the gradual progression of training volume and were instructed to report any recurrence or increase in pain in the affected tibial region. Because the participants represented different sports with distinct physical and technical demands, the subsequent return-to-training and return-to-sport progression was individualized and managed by each participant’s coach. Participants were advised to resume sport-specific training at an initially reduced load, with subsequent progression based on the demands of the respective sport and their symptom response. Sport-specific exercises, training volume, and progression criteria were therefore not standardized by the research team. Training load and adherence during this 12-week period were not systematically monitored. Return-to-sport timing was not a prespecified study outcome and was not systematically recorded using a standardized definition.

#### 2.5.2. Physiotherapy Rehabilitation Program Plus Functional Magnetic Stimulation (FMS)

**Figure 1:**
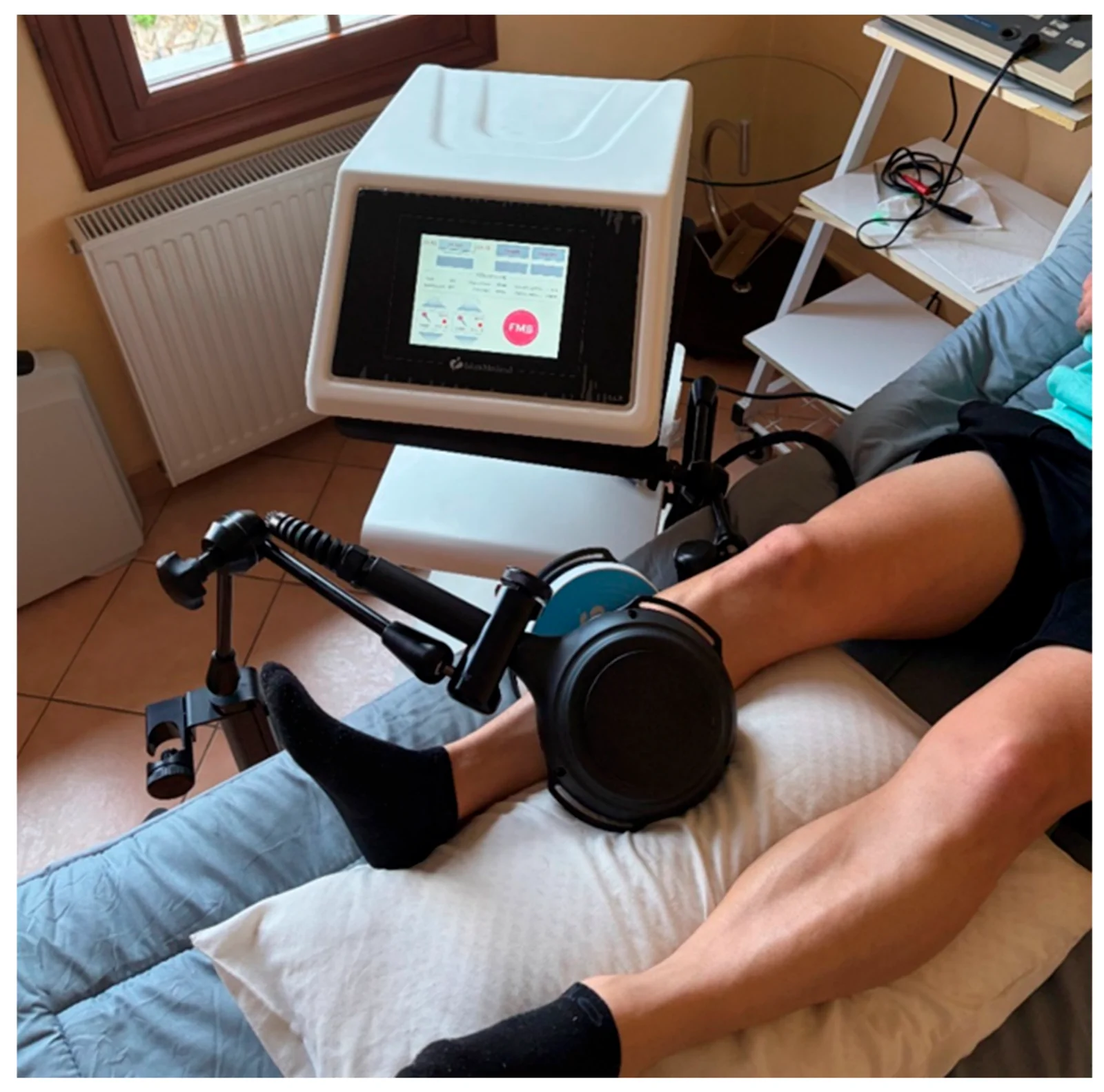
Functional Magnetic Stimulation (FMS) application over the tibial region.

Participants allocated to the intervention group received FMS in addition to the standardized physiotherapy program. FMS was delivered twice weekly for four weeks, resulting in a total of eight sessions, with each session lasting 30 min. Treatment was administered using a clinical magnetic stimulation device (Tesla Stym, Iskra Medical, Ljubljana, Slovenia) over the affected tibial region. According to the manufacturer-confirmed technical specifications, the device generated biphasic electromagnetic pulses with a pulse duration of 0.3 ms and had a maximum nominal magnetic flux density of 3 Tesla (T). This value represents the maximum output capability of the device rather than the participant-specific treatment dose, which was not recorded as an absolute magnetic field strength. A bipolar application was used, with two round L applicators, each 16 cm in diameter, positioned simultaneously around the symptomatic tibial segment to encompass the clinically involved area (Figure 1). Stimulation was delivered at a fixed frequency of 40 Hz without modulation, using a duty cycle of 3 s on and 6 s off, in accordance with the predefined treatment protocol. The 40 Hz setting corresponded to a manufacturer-preconfigured protocol; however, no FMS-specific dose–response evidence has established this frequency as optimal for TBSI or BME. Stimulation intensity was progressively increased to the maximum level tolerated by the participant while maintaining comfort and avoiding pain provocation or excessive discomfort. FMS was applied as an adjunctive modality and was not intended to replace the loading-based rehabilitation approach, but rather to complement the physiotherapy program within the same treatment period. Participants in the control group received the same physiotherapy program without FMS.

#### 2.5.3. Adverse Event Monitoring

Adverse events were actively monitored in both groups throughout the 4-week supervised intervention period. At each supervised treatment session, participants were systematically asked before and after treatment about any new or worsening symptoms, including increased tibial pain, muscle discomfort, cramping, dizziness, skin irritation, or any other unexpected complaint. Any adverse event reported spontaneously or elicited through structured questioning was documented by the treating physiotherapist.

### 2.6. Sample Size Estimation

An a priori sample size calculation was performed using G*Power software, version 3.1.9.7. The calculation was aligned with the planned repeated-measures analysis of the primary clinical outcome. The calculation was based on an F-test for repeated-measures ANOVA with a within–between interaction, using two groups and three repeated measurements. The input parameters were set as follows: effect size f = 0.25, α error probability = 0.05, statistical power = 0.90, number of groups = 2, number of measurements = 3, correlation among repeated measures = 0.50, and nonsphericity correction ε = 1. The analysis indicated that a total sample size of 36 participants would be required to achieve an actual power of 0.906. To account for potential dropout during follow-up, four additional participants were included. Therefore, the final target sample size was set at 40 participants, with 20 participants allocated to each group.

### 2.7. Statistical Analysis

Statistical analyses were performed using IBM SPSS Statistics for Windows, version 25.0 (IBM Corp., Armonk, NY, USA). All analyses were conducted according to the intention-to-treat principle, with all randomized participants analyzed in the groups to which they were originally allocated. Because all randomized participants completed follow-up and were included in the analyses, no outcome data were missing and no imputation was required. Descriptive statistics were used to summarize demographic and baseline clinical characteristics of the sample. Continuous variables are presented as mean ± standard deviation, ordinal variables as median (Q1–Q3) and frequencies and percentages, and categorical variables as frequencies and percentages, as appropriate. The normality of continuous variables was assessed using the Shapiro–Wilk test and visual inspection of Q–Q plots.

Between-group differences in baseline characteristics were examined to assess baseline comparability between groups. Independent-samples *t*-tests were used for normally distributed continuous variables, the Mann–Whitney U test for non-normally distributed continuous variables, and chi-square tests or Fisher’s exact tests for categorical variables, as appropriate.

The primary analysis examined the group × time interaction for activity-related pain (NPRS). Secondary analyses examined lower-limb functional ability (LEFS-GR), MRI-derived BME extent, and Fredericson MRI grade. The NPRS group × time interaction was the confirmatory primary-outcome analysis. The analyses of LEFS-GR, BME extent, and Fredericson grade were considered exploratory secondary-outcome analyses. No multiplicity adjustment was applied across the outcome-level interaction tests; therefore, the secondary-outcome findings should be interpreted as exploratory and supportive. Bonferroni adjustment was applied to the post hoc pairwise comparisons conducted for the continuous outcomes. When the group × time interaction for Fredericson grade was significant, time-specific between-group contrasts were obtained by changing the reference time point in the same ordinal GEE model and were reported as cumulative ORs with 95% confidence intervals. These exploratory contrasts were not adjusted for multiplicity. The effects of the intervention on repeated-measures continuous outcomes, including activity-related pain (NPRS), lower-limb functional ability (LEFS-GR), and MRI-derived BME extent, were analyzed using two-way mixed analysis of variance (mixed ANOVA), with group (intervention vs. control) as the between-subjects factor and time (baseline, 4 weeks, and 16 weeks) as the within-subjects factor. The group × time interaction term was used to determine whether changes over time differed between groups. When a significant group × time interaction was observed, post hoc simple effects analyses were performed using Bonferroni-adjusted pairwise comparisons of estimated marginal means to identify between-group differences at each time point and within-group changes over time. Mauchly’s test was used to assess the assumption of sphericity, and Greenhouse–Geisser correction was applied when this assumption was violated. Effect sizes for ANOVA models were reported as partial eta squared (η^2^p).

The Fredericson MRI grade, as an ordinal repeated-measures outcome, was analyzed separately using an ordinal generalized estimating equation (GEE) model with a cumulative logit link. The model included group, time, and the group × time interaction as model effects, with participant ID specified as the subject variable and time as the within-subject factor. An exchangeable working correlation structure and robust covariance estimator were used. Model coefficients were exponentiated and reported as cumulative odds ratios (ORs) with 95% confidence intervals. Because higher Fredericson grades indicate greater MRI severity, ORs below 1 indicate lower cumulative odds of classification in a higher severity grade. The group × time interaction was considered the main effect of interest for determining whether changes in MRI severity classification differed between groups over time. Fredericson grades were also presented descriptively as median (Q1–Q3) and as frequencies and percentages for each grade at each assessment time point.

For the post-hoc reliability analysis, intra-rater and inter-rater reliability of the linear BME extent measurements were assessed separately at baseline, 4 weeks, and 16 weeks using intraclass correlation coefficients (ICCs) with 95% confidence intervals. Intra-rater reliability was calculated using a two-way mixed-effects, absolute-agreement, single-measure model, whereas inter-rater reliability was calculated using a two-way random-effects, absolute-agreement, single-measure model.

All statistical tests were two-tailed, and the level of statistical significance was set at *p* < 0.05.

## 3. Results

**Figure 2:**
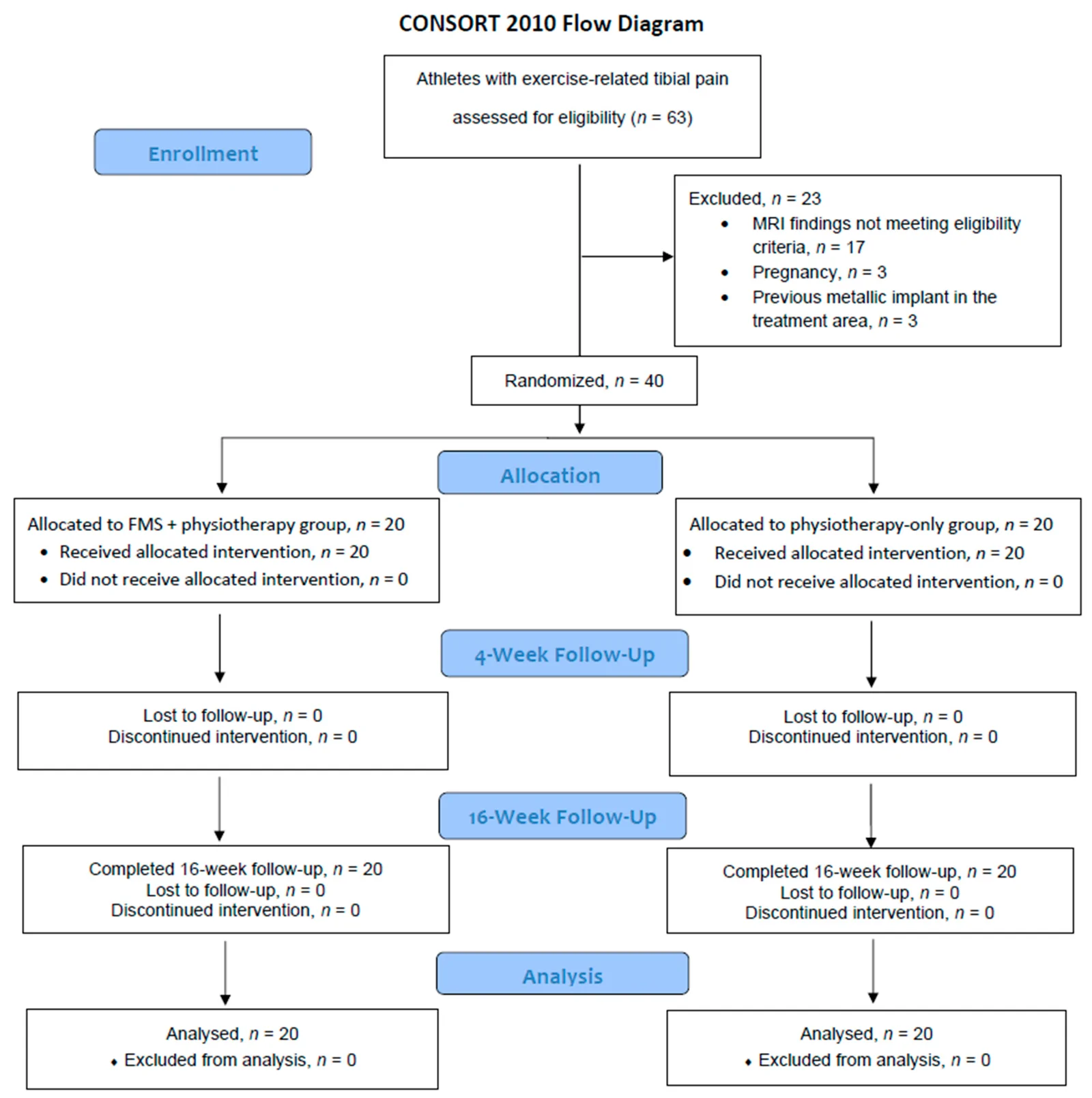
CONSORT flow diagram of participant recruitment, randomization, follow-up, and analysis.

A total of 63 athletes with exercise-related tibial pain were assessed for eligibility between December 2025 and February 2026. Of these, 23 were excluded for not meeting the predefined eligibility criteria, as detailed in the CONSORT flow diagram. The remaining 40 athletes were enrolled and randomized, with 20 participants allocated to the intervention group and 20 to the control group. All randomized participants completed the baseline, 4-week, and 16-week follow-up assessments. No participants were lost to follow-up, and no dropouts occurred during the study period. No adverse events or intervention-related symptoms were identified through structured questioning or spontaneous reporting in either group during the 4-week supervised intervention period. The participant flow through the study is presented in Figure 2.

Baseline demographic and sport-related characteristics of the participants are presented in Table 1. No statistically significant between-group differences were observed for demographic, sport-related, or baseline MRI severity characteristics, indicating that the two groups were comparable at baseline.

### 3.1. Primary Outcome: Activity-Related Pain During Standardized Treadmill Walking (NPRS)

For NPRS scores, a significant group × time interaction was found, F(2, 76) = 12.46, *p* < 0.001, η^2^p = 0.247. Significant main effects of time, F(2, 76) = 217.21, *p* < 0.001, η^2^p = 0.851, and group, F(1, 38) = 23.23, *p* < 0.001, η^2^p = 0.379, were also observed. Bonferroni-adjusted simple effects analyses showed no significant between-group difference at baseline (*p* = 0.702), whereas significant between-group differences were identified at 4 weeks (mean difference = −1.55, 95% CI: −2.11 to −0.99, *p* < 0.001) and 16 weeks (mean difference = −0.60, 95% CI: −0.93 to −0.27, *p* = 0.001), with lower NPRS scores in the intervention group. Descriptive values are presented in Table 2.

### 3.2. Secondary Outcome: Lower-Limb Functional Ability (LEFS-GR)

For LEFS-GR scores, a significant group × time interaction was found, F(1.08, 41.09) = 81.56, *p* < 0.001, η^2^p = 0.682. Significant main effects of time, F(1.08, 41.09) = 1419.86, *p* < 0.001, η^2^p = 0.974, and group, F(1, 38) = 22.62, *p* < 0.001, η^2^p = 0.373, were also observed. Bonferroni-adjusted simple effects analyses showed no significant between-group difference at baseline (*p* = 0.311), whereas significant between-group differences were identified at 4 weeks (mean difference = 6.80, 95% CI: 4.68 to 8.92, *p* < 0.001) and 16 weeks (mean difference = 6.65, 95% CI: 5.30 to 8.00, *p* < 0.001), with higher LEFS-GR scores in the intervention group. Descriptive values are presented in Table 2.

### 3.3. Secondary Outcome: Bone Marrow Edema (BME) Extent

For BME extent, a significant group × time interaction was found, F(1.44, 54.85) = 49.33, *p* < 0.001, η^2^p = 0.565. Significant main effects of time, F(1.44, 54.85) = 2403.42, *p* < 0.001, η^2^p = 0.984, and group, F(1, 38) = 7.20, *p* = 0.011, η^2^p = 0.159, were also observed. Bonferroni-adjusted simple effects analyses showed no significant between-group difference at baseline (*p* = 0.557), whereas significant between-group differences were identified at 4 weeks (mean difference = −0.71, 95% CI: −1.02 to −0.40, *p* < 0.001) and 16 weeks (mean difference = −0.93, 95% CI: −1.35 to −0.51, *p* < 0.001), with lower BME extent in the intervention group. Descriptive values are presented in Table 2.

The post-hoc reliability analysis included MRI examinations from the same 30 participants at each assessment time point. Intra-rater reliability for the linear BME extent measurements was excellent, with single-measure absolute-agreement ICCs of 0.981 (95% CI: 0.961–0.991) at baseline, 0.973 (95% CI: 0.944–0.987) at 4 weeks, and 0.970 (95% CI: 0.939–0.986) at 16 weeks. Inter-rater reliability was also excellent, with corresponding ICCs of 0.910 (95% CI: 0.820–0.956), 0.962 (95% CI: 0.887–0.984), and 0.965 (95% CI: 0.929–0.983), respectively. All ICCs were statistically significant (*p* < 0.001).

### 3.4. Secondary Outcome: Fredericson Grade (MRI Severity Classification)

Fredericson grade was analyzed as an ordinal repeated-measures outcome using a generalized estimating equation model with a cumulative logit link. A significant group × time interaction was found, Wald χ^2^(2) = 20.38, *p* < 0.001, indicating that changes in MRI severity classification over time differed between groups. Significant main effects of time, Wald χ^2^(2) = 31.40, *p* < 0.001, and group, Wald χ^2^(1) = 6.25, *p* = 0.012, were also observed. At the 4-week follow-up, the intervention group had significantly lower cumulative odds of being classified in a higher Fredericson grade than the control group (OR = 0.045, 95% CI: 0.012–0.163, *p* < 0.001), corresponding to approximately 95.5% lower odds of greater MRI severity. At the 16-week follow-up, the corresponding difference remained directionally favorable to the intervention group but did not reach statistical significance (OR = 0.033, 95% CI: 0.001–1.029, *p* = 0.052). Descriptively, Fredericson grades shifted toward lower severity in both groups, with a more favorable pattern in the intervention group. At the 16-week follow-up, 4/20 participants (20.0%) in the intervention group reached grade 0 compared with 0/20 participants in the control group, while no participant in the intervention group remained at grade 3 compared with 3/20 participants (15.0%) in the control group. Descriptive values are presented in Table 2.

## 4. Discussion

The present randomized controlled trial examined the effects of adding FMS to a standardized physiotherapy rehabilitation program in athletes with MRI-confirmed tibial bone stress injury. The present study evaluated clinical, functional, and MRI-derived outcomes across baseline, 4 weeks, and a 16-week follow-up, thereby allowing assessment of both early and medium-term recovery patterns. The findings indicate that the integration of FMS was associated with greater improvement in the primary outcome of activity-related pain, as well as improvements in the secondary outcomes of lower-limb functional ability and MRI-derived indicators of lesion severity and bone marrow edema extent, compared with rehabilitation alone. Significant group × time interactions were observed for the primary outcome (NPRS) and for the secondary outcomes of LEFS-GR, BME extent, and Fredericson grade, indicating that the trajectory of recovery differed between groups over the 16-week follow-up period. These results extend current evidence on conservative management of bone stress injuries, where recovery is often prolonged and variable despite commonly recommended load-management strategies, and where the role of adjunctive non-invasive interventions remains insufficiently defined [6,9,11]. Within this context, the present study provides preliminary controlled randomized evidence suggesting that FMS may enhance clinical, functional, and MRI-derived recovery when incorporated into a structured, load-based rehabilitation approach.

To place the primary NPRS findings in a clinical context, Fleagle et al. [25] reported a minimal clinically important change of 1.1 points on a 0–10 scale for pain measured during clinically relevant movement tasks. In the present study, the between-group difference in treadmill-provoked tibial pain was 1.55 points at 4 weeks, exceeding this external reference value and suggesting that the early advantage associated with FMS may have been clinically meaningful. However, the lower bound of the 95% confidence interval was 0.99 points and therefore slightly below this benchmark. In contrast, the between-group difference at 16 weeks was 0.60 points and did not exceed this reference value, despite remaining statistically significant. The threshold reported by Fleagle et al. was derived from individuals with fibromyalgia and chronic Achilles tendinopathy and represents a within-person minimal clinically important change rather than a condition-specific between-group MCID for athletes with TBSI. Therefore, this comparison should be interpreted as contextual evidence of possible clinical relevance rather than definitive confirmation of a clinically important treatment effect.

A similar distinction between statistical and clinical significance is required for the LEFS-GR findings. The original development study of the LEFS reported a 9-point change as a reference for clinically meaningful functional improvement [22]. In the present study, the between-group differences were 6.80 points at 4 weeks and 6.65 points at 16 weeks. Although both differences were statistically significant, neither exceeded this external reference value. Therefore, the additional functional advantage associated with FMS cannot be considered definitively clinically important on the basis of the between-group mean differences alone. Descriptively, the mean improvement from baseline in the intervention group was 11.47 points at 4 weeks and 22.67 points at 16 weeks, whereas the corresponding changes in the control group were 3.55 and 14.90 points. This pattern suggests that meaningful functional improvement may have occurred earlier in the intervention group, while substantial improvement was observed in both groups by 16 weeks. However, the 9-point threshold was derived from a broader lower-extremity musculoskeletal population, has not been specifically validated in athletes with TBSI, and primarily represents an individual-level change threshold rather than a condition-specific between-group MCID. Moreover, because no responder analysis was prespecified, the proportion of individual participants who achieved clinically meaningful functional improvement cannot be determined.

The greater improvements observed in activity-related pain and lower-limb functional ability in the intervention group may reflect, at least in part, greater rehabilitation tolerance and more effective participation in progressive loading, potentially facilitated by the analgesic and neuromuscular effects proposed for FMS in other rehabilitation contexts [16,17,18]. Pain in tibial bone stress injury is closely related to repetitive loading and the accumulation of microdamage, while biomechanical and neuromuscular factors influence how mechanical stress is transferred to and tolerated by the tibia during activity [3,6]. Within this framework, the addition of FMS may have supported participation in the progressive loading phases of rehabilitation by reducing activity-related symptoms and improving perceived tolerance to loading, without implying a direct bone-healing effect. The significant between-group differences observed at both 4 weeks and 16 weeks suggest that the benefits associated with FMS persisted beyond the immediate intervention period and were still evident at medium-term follow-up. This interpretation is consistent with contemporary rehabilitation approaches for tibial bone stress injury, which emphasize symptom-guided graded loading, progressive return to activity, and the role of strength and biomechanical factors in restoring load tolerance [9]. The observed improvements in LEFS-GR scores further support this interpretation, suggesting that the intervention was associated not only with symptom reduction but also with improved self-reported lower-limb functional ability relevant to daily and basic sport-related activities.

The observed reductions in BME extent and the improvement in Fredericson grade in the intervention group suggest that the addition of FMS may be associated not only with clinical improvement but also with more favorable MRI-derived changes in lesion status. In tibial bone stress injury, bone marrow edema detected on MRI is considered an important imaging feature of the injury spectrum, reflecting abnormal marrow signal changes associated with osseous stress response, increased bone turnover, and disruption in the balance between microdamage formation and repair [3,5,6,7]. Although structural recovery is typically thought to occur more gradually than symptom resolution, the present findings indicate that measurable improvements in MRI-derived parameters occurred over the 16-week follow-up period, with greater reductions in BME extent and a more favorable shift in Fredericson classification in the intervention group. MRI-based grading and semiquantitative MRI features of tibial stress injury have been associated with injury severity and time to return to sport, although the prognostic distinction between intermediate grades may be limited [26]. The between-group difference in Fredericson grade was statistically significant at 4 weeks, whereas at the 16-week follow-up the corresponding cumulative odds remained directionally favorable to the intervention group but did not reach statistical significance. Descriptively, 4/20 participants (20.0%) in the intervention group reached grade 0 at 16 weeks compared with 0/20 participants in the control group, while no participant in the intervention group remained at grade 3 compared with 3/20 participants (15.0%) in the control group. Importantly, MRI-derived improvement was observed over time in both groups, indicating that part of the reduction in BME extent and Fredericson grade likely reflected the expected temporal recovery of the injury, load modification, and participation in the 4-week standardized rehabilitation program. The significant group × time interactions indicate that the trajectory of MRI-derived recovery differed between groups, but they do not establish that FMS directly altered bone remodeling or independently accelerated biological healing. Because the study did not include an untreated natural-history comparison group, and because training exposure and loading progression between weeks 4 and 16 were not systematically standardized, the relative contributions of temporal recovery, rehabilitation, subsequent activity, and FMS cannot be fully separated. Accordingly, the MRI findings should be interpreted as exploratory imaging correlates of a more favorable recovery trajectory associated with the addition of FMS, rather than as definitive evidence of accelerated or complete biological bone healing.

The potential mechanisms underlying the observed effects of FMS may be interpreted in the context of electromagnetic stimulation and its interaction with neuromuscular and biological tissue processes. FMS delivers time-varying magnetic fields that induce electric currents within excitable tissues, leading to depolarization of peripheral nerves and subsequent muscle contractions, and may also influence sensory pathways indirectly [16,17]. Through these mechanisms, FMS may contribute to pain modulation, neuromuscular activation, and improved rehabilitation tolerance, factors that are considered relevant in musculoskeletal rehabilitation and have been suggested in other FMS rehabilitation contexts [16,17,18]. In the present study, these effects may have supported the rehabilitation process indirectly by reducing pain-related inhibition, improving perceived loading tolerance, and allowing more effective progression through the structured exercise program. In addition, although direct evidence in bone stress injury is limited, related forms of electromagnetic stimulation, particularly pulsed electromagnetic fields, have been investigated in bone marrow edema conditions and have been associated with improvements in pain, function, and, in some cases, MRI findings [13,14,15]. These findings provide only indirect biological and clinical rationale and should not be interpreted as direct evidence that FMS accelerates bone remodeling or edema resolution in TBSI. Nevertheless, these biological mechanisms remain hypothetical in the context of tibial bone stress injury and require confirmation in future mechanistic studies.

These considerations are consistent with the broader literature on non-invasive biophysical interventions. Extracorporeal shock wave therapy (ESWT) has been reported to improve pain and functional outcomes in athletes with bone stress injuries, although the available evidence remains limited and is derived largely from retrospective studies, case series, and broader reviews of adjunctive treatment options [4,12]. Similarly, pulsed electromagnetic fields (PEMFs) have been associated with improvements in pain, function, and, in some cases, MRI findings in conditions involving bone marrow edema, although the available studies are heterogeneous in terms of patient population, anatomical region, and underlying diagnosis [13,14,15]. Within this context, FMS may be viewed as a related but distinct modality, combining electromagnetic stimulation with neuromuscular activation, and may therefore provide a complementary pathway for influencing both functional and tissue-level aspects of recovery. The present findings add to this field by providing randomized controlled evidence in a specific athletic population with MRI-confirmed tibial bone stress injury and by demonstrating benefits that persisted beyond the immediate post-intervention period.

From a clinical perspective, the findings of the present study suggest that the addition of FMS to a structured physiotherapy program may represent a potentially useful adjunct in the management of athletes with tibial bone stress injury. Contemporary rehabilitation approaches emphasize symptom-guided load management and progressive reloading as central components of recovery, with the goal of restoring load tolerance while minimizing the risk of symptom exacerbation [6,9]. Within this framework, interventions that can reduce pain and improve functional capacity may facilitate more effective engagement in rehabilitation processes and support the progression of graded loading strategies. The persistence of statistically significant between-group differences in pain and function at 16 weeks suggests that the addition of FMS may have been associated with a more favorable recovery trajectory over time, although the magnitude of the between-group differences in pain and function did not exceed the external reference values discussed above.

The observed improvements in pain and function in the intervention group suggest that FMS may have contributed to this process by supporting greater perceived and functional tolerance to mechanical loading during rehabilitation. This may be particularly relevant in the early phases of recovery, where pain-related limitations often restrict progression and delay return to activity. At the same time, the findings related to MRI outcomes suggest that FMS may not only influence symptom perception but also be associated with favorable MRI-derived changes in lesion status, although this requires further confirmation. Importantly, the parallel improvement in clinical, functional, and MRI-derived outcomes supports the interpretation that the observed effects were not limited to subjective symptom relief, but were compatible with a broader recovery pattern.

Importantly, FMS should not be considered a standalone treatment but rather an adjunct to established rehabilitation principles. Load management, progressive strengthening, and correction of contributing factors remain central components of treatment in bone stress injuries, and the role of FMS should be viewed within a multimodal approach aimed at optimizing both clinical and functional outcomes. Although MRI-derived outcomes were more favorable in the intervention group, these exploratory findings should not be interpreted as definitive evidence that FMS accelerates biological bone healing. The present findings therefore support considering FMS as a complementary modality within a structured rehabilitation framework, rather than as a replacement for progressive loading and individualized return-to-activity planning.

Several limitations of the present study should be considered when interpreting the findings. First, although the sample size was determined a priori and the study was conducted as a randomized controlled trial, the total sample remained modest and was drawn from a specific athletic population, which may limit the generalizability of the results. In addition, the study was conducted in a single research setting, and therefore the findings should be confirmed in larger multicenter trials. Symptom duration before enrollment was not prospectively recorded with sufficient consistency, preventing assessment of baseline injury chronicity and its potential influence on rehabilitation response or MRI-derived recovery.

Second, although the 16-week follow-up provides more information than an immediate post-intervention assessment, it does not allow conclusions regarding long-term recurrence risk, full return-to-sport timelines, or the durability of MRI recovery beyond this period. Importantly, improvement in BME extent or Fredericson grade should not be interpreted as evidence of complete biological healing or as confirmation of readiness for unrestricted return to sport. In the absence of prospectively defined return-to-sport criteria integrating symptoms, functional performance, and progressive load tolerance, MRI improvement alone cannot establish readiness for safe, unrestricted return to sport. Given that bone stress injuries may require prolonged structural adaptation and careful reintegration into high-impact sport, longer follow-up is necessary to determine the persistence and clinical relevance of the observed changes.

Third, the return-to-training period between weeks 4 and 16 was not standardized or systematically monitored by the research team. Although all participants received the same general written guidance regarding gradual increases in training volume and the reporting of recurrent tibial pain, sport-specific progression was individualized and largely managed by the participants and their respective coaches. Consequently, differences in training exposure, loading progression, adherence, or additional sport-specific exercise between groups may have influenced the LEFS-GR and MRI outcomes observed at the 16-week reassessment and cannot be excluded as potential confounders. Moreover, sufficiently reliable descriptive data on return-to-sport timing were not available for inclusion in the present analysis. Future trials should prospectively define and systematically record return-to-sport milestones, including return to modified training, full training, and unrestricted competition.

Fourth, blinding of participants and treating physiotherapists was not feasible due to the nature of the intervention, which may introduce performance bias. More importantly, the absence of a sham-FMS arm and the resulting inability to blind participants represent a major limitation and a threat to internal validity, particularly for the self-reported NPRS and LEFS-GR outcomes. Expectancy, additional therapeutic attention, and other contextual effects may therefore have contributed to the observed between-group differences in pain and function. Although outcome assessment was performed by a blinded assessor, the effects observed in these self-reported outcomes cannot be attributed exclusively to the specific physiological action of FMS. The MRI-derived outcomes were less directly susceptible to participant expectations and were evaluated by a blinded assessor; nevertheless, the patient-reported findings should be interpreted cautiously pending confirmation in sham-controlled trials.

Fifth, although the post-hoc reliability analysis demonstrated excellent intra-rater and inter-rater reliability for the BME extent measurements, BME extent was quantified as the maximum longitudinal extent observed on a single sagittal MRI slice. This linear measurement represents a relatively coarse surrogate of overall edema burden and does not capture lesion width, depth, or total volume. Therefore, the demonstrated reproducibility should not be interpreted as equivalence to a comprehensive volumetric assessment of BME. Future studies should consider validated semiquantitative approaches or three-dimensional volumetric segmentation to provide a more comprehensive evaluation of changes in edema extent.

Sixth, several of the partial eta-squared estimates observed in the present study were unusually large for an adjunctive rehabilitation intervention and should therefore be interpreted cautiously. In particular, the η^2^p value of 0.984 for BME extent represents the overall main effect of time across both groups and should not be interpreted as a treatment-specific effect of FMS. Nevertheless, the magnitude of the reported effect sizes may partly reflect the modest sample size, the single-center design, and the use of a single primary assessor for the original MRI outcome evaluation, despite the excellent reliability demonstrated in the post-hoc analysis. For the NPRS outcome, the use of a standardized, relatively low-impact treadmill walking task may also have reduced measurement variability compared with more demanding sport-specific loading. These effect-size estimates require confirmation in larger multicenter trials with multiple independent MRI assessors and more sport-specific functional testing.

Seventh, the 10 min treadmill walking protocol was adapted from running-based protocols and was not formally pilot-tested or evaluated for test–retest reliability before the trial. Although the procedure was standardized across assessment time points by maintaining the same individualized walking speed for each participant, the use of a self-selected speed may have introduced between-participant variability in mechanical loading and symptom provocation. Consequently, the validity, reproducibility, and responsiveness of this protocol as an outcome-assessment method remain uncertain. Future studies should formally evaluate its measurement properties or employ a validated and clinically appropriate activity-based loading test.

Finally, the study population consisted of relatively young and physically active athletes with moderate-severity tibial bone stress injury, corresponding to Fredericson grades 2–3. This may limit the applicability of the findings to other populations, such as non-athletic individuals, military recruits, older patients, or athletes with more advanced lesions. Future multicenter trials with larger samples, longer follow-up periods, sham-controlled designs, and direct return-to-sport outcomes are needed to confirm these findings and to clarify whether the observed improvements translate into faster and safer return to full athletic participation.

## 5. Conclusions

The findings of this randomized controlled trial suggest that the addition of FMS to a standardized 4-week, load-based rehabilitation program was associated with greater improvements in activity-related pain, lower-limb functional ability, bone marrow edema extent, as well as a more favorable longitudinal trajectory in MRI severity classification than rehabilitation alone over the 16-week follow-up period. However, because no sham-FMS arm was included and participants could not be blinded, the observed between-group differences in the self-reported pain and functional outcomes cannot be attributed with certainty to the specific physiological effects of FMS. FMS should therefore be considered a potentially useful adjunct to, rather than a replacement for, progressive load management and individualized rehabilitation. Further studies with larger samples, sham-controlled designs, longer follow-up periods, and direct return-to-sport outcomes are needed to confirm these findings and better define the clinical role of FMS in the management of bone stress injuries.

## Figures and Tables

**Table 1:** Baseline demographic and sport-related characteristics.

Variable	Intervention (*n* = 20)	Control (*n* = 20)	Between-Group *p*-Value
Age (years), mean ± SD	26.67 ± 4.62	27.65 ± 4.82	0.514 ^a^
Height (cm), mean ± SD	175.50 ± 9.24	176.00 ± 13.06	0.890 ^a^
Weight (kg), mean ± SD	70.83 ± 11.98	75.25 ± 14.25	0.295 ^a^
BMI (kg/m^2^), mean ± SD	22.86 ± 2.58	24.08 ± 2.92	0.169 ^a^
Training age (months), mean ± SD	46.09 ± 15.25	42.89 ± 8.31	0.417 ^a^
Sex, *n* (%)			
Male	9 (45.0)	10 (50.0)	0.752 ^b^
Female	11 (55.0)	10 (50.0)	
Leg dominance, *n* (%)			
Right	17 (85.0)	18 (90.0)	1.000 ^c^
Left	3 (15.0)	2 (10.0)	
Affected side, *n* (%)			
Right	13 (65.0)	13 (65.0)	1.000 ^b^
Left	7 (35.0)	7 (35.0)	
Sport, *n* (%)			
Endurance running/track & field	11 (55.0)	10 (50.0)	0.943 ^d^
Soccer (football)	6 (30.0)	5 (25.0)	
Basketball	1 (5.0)	3 (15.0)	
Volleyball	1 (5.0)	1 (5.0)	
CrossFit/HIIT/functional training	1 (5.0)	1 (5.0)	
Fredericson grade at baseline, *n* (%)			
Grade 2	11 (55.0)	11 (55.0)	1.000 ^b^
Grade 3	9 (45.0)	9 (45.0)	

Abbreviations: BMI = body mass index; HIIT = high-intensity interval training; *n* = number of participants; SD = standard deviation. ^a^ Independent-samples *t*-test; ^b^ Chi-square test; ^c^ Fisher’s exact test; ^d^ Fisher–Freeman–Halton exact test.

**Table 2:** Changes in Clinical and MRI Outcomes Across Baseline, 4 Weeks, and 16 Weeks.

Group	Baseline	Week 4	16-Week Follow-Up	Between-Group Post Hoc Simple Effects †	Time Effect/Model Effect	Interaction *p*-Value
Activity-related pain (NPRS)						
Intervention	3.95 ± 0.83	1.20 ± 0.95	0.15 ± 0.37	Baseline: *p* = 0.702 Week 4: MD = −1.55, 95% CI: −2.11 to −0.99, *p* < 0.001 16-week follow-up: MD = −0.60, 95% CI: −0.93 to −0.27, *p* = 0.001	F(2, 76) = 217.21 *p* < 0.001 η^2^p = 0.851	<0.001
Control	3.85 ± 0.81	2.75 ± 0.79	0.75 ± 0.64			
Lower-limb function (LEFS-GR)						
Intervention	54.88 ± 3.37	66.35 ± 3.47	77.55 ± 2.01	Baseline: *p* = 0.311 Week 4: MD = 6.80, 95% CI: 4.68 to 8.92, *p* < 0.001 16-week follow-up: MD = 6.65, 95% CI: 5.30 to 8.00, *p* < 0.001	F(1.08, 41.09) = 1419.86 *p* < 0.001 η^2^p = 0.974	<0.001
Control	56.00 ± 3.55	59.55 ± 3.15	70.90 ± 2.20			
Bone marrow edema extent (cm)						
Intervention	5.31 ± 0.74	3.31 ± 0.48	0.89 ± 0.58	Baseline: *p* = 0.557 Week 4: MD = −0.71, 95% CI: −1.02 to −0.40, *p* < 0.001 16-week follow-up: MD = −0.93, 95% CI: −1.35 to −0.51, *p* < 0.001	F(1.44, 54.85) = 2403.42, *p* < 0.001, η^2^p = 0.984	<0.001
Control	5.18 ± 0.68	4.02 ± 0.50	1.82 ± 0.73			
Fredericson grade						
Intervention	2 (2–3)	2 (2–2)	2 (1.25–2)	Week 4: OR = 0.045, 95% CI: 0.012–0.163, *p* < 0.001 16-week follow-up: OR = 0.033, 95% CI: 0.001–1.029, *p* = 0.052	Wald χ^2^(2) = 31.40, *p* < 0.001 Group: Wald χ^2^(1) = 6.25, *p* = 0.012	<0.001
Control	2 (2–3)	2 (2–3)	2 (2–2)			

Abbreviations: NPRS, Numeric Pain Rating Scale; LEFS-GR, Greek version of the Lower Extremity Functional Scale; BME, bone marrow edema; MRI, magnetic resonance imaging; OR, odds ratio; SD, standard deviation; MD, mean difference; CI, confidence interval; Q1–Q3, first to third quartile. Values are presented as mean ± SD for NPRS, LEFS-GR, and BME extent, and as median (Q1–Q3) for Fredericson grade. Continuous outcomes were analyzed using two-way mixed ANOVA; interaction *p*-values refer to the group × time effect. Greenhouse–Geisser-corrected values are reported where applicable. Bonferroni-adjusted simple effects represent between-group comparisons at each time point and are expressed as intervention minus control. Fredericson grade was analyzed using an ordinal generalized estimating equation model with a cumulative logit link; lower grades indicate lower MRI severity. † For NPRS, LEFS-GR, and BME extent, between-group post hoc simple effects were based on Bonferroni-adjusted comparisons of estimated marginal means at each assessment time point. For Fredericson grade, time-specific between-group comparisons were derived from the ordinal GEE model and reported as cumulative ORs with 95% CIs; these exploratory contrasts were not multiplicity-adjusted.

## Data Availability

The data that support the findings of this study are openly available in Zenodo at https://doi.org/10.5281/zenodo.21301621.

## Supplementary Materials

The following supporting information can be downloaded at: https://www.mdpi.com/article/10.3390/jfmk11030317/s1, Table S1: Standardized Physiotherapy Rehabilitation Program; File S1: CONSORT 2010 Checklist.
