# Supplementary material for: Effects of Functional Magnetic Stimulation on Pain, Function, and MRI-Derived Outcomes in Athletes with Tibial Bone Stress Injury: A Randomized Controlled Trial
Source: J Funct Morphol Kinesiol. 2026 Aug 14;11(3):317. doi: 10.3390/jfmk11030317 (PMC13511723; doi:10.3390/jfmk11030317)
Supplement: Supplementary file 1 [file jfmk-11-00317-s001.zip › CONSORT_Checklist_JFMK_Lytras_2026 revised.pdf]

CONSORT 2010 Checklist

Completed checklist for manuscript submission

Manuscript title: Effects of Functional Magnetic Stimulation on Pain, Function, and MRI-Derived Outcomes in Athletes with Tibial Bone Stress Injury: A Randomized Controlled Trial

Trial design: Two-arm, parallel-group randomized controlled trial with blinded outcome assessment, three assessment time points, and 1:1 allocation ratio.

Trial registration: ClinicalTrials.gov, NCT07234084.

Ethics approval: Ethics Committee of the Department of Physiotherapy, Faculty of Health Sciences, International Hellenic University, EC-15/2025.

Section/Topic	Item No.	Checklist item	Reported on page No.
**Title and Abstract**	1a	Identification as a randomized trial in the title.	1
	1b	Structured summary of trial design, methods, results, and conclusions.	1-2
**Introduction: Background and objectives**	2a	Scientific background and explanation of rationale.	2-4
	2b	Specific objectives or hypotheses.	4
**Methods: Trial design**	3a	Description of trial design, including allocation ratio.	4
	3b	Important changes to methods after trial commencement, with reasons.	Not applicable; no important changes were made after trial commencement.
**Methods: Participants**	4a	Eligibility criteria for participants.	5
	4b	Settings and locations where the data were collected.	4
**Methods: Interventions**	5	The interventions for each group with sufficient details to allow replication, including how and when they were actually administered.	7-9; Supplementary Table S1
**Methods: Outcomes**	6a	Completely defined pre-specified primary and secondary outcome measures, including how and when they were assessed.	5-7
	6b	Any changes to trial outcomes after the trial commenced, with reasons.	Not applicable; no changes were made to the pre-specified trial outcomes.
**Methods: Sample size**	7a	How sample size was determined.	9
	7b	When applicable, explanation of any interim analyses and stopping guidelines.	Not applicable; no interim analyses were planned or performed.
**Methods: Randomization - sequence generation**	8a	Method used to generate the random allocation sequence.	4
	8b	Type of randomization; details of any restriction, such as blocking and block size.	4

Section/Topic	Item No.	Checklist item	Reported on page No.
**Methods: Randomization - allocation concealment mechanism**	9	Mechanism used to implement the random allocation sequence, describing any steps taken to conceal the sequence until interventions were assigned.	4
**Methods: Randomization - implementation**	10	Who generated the random allocation sequence, who enrolled participants, and who assigned participants to interventions.	4
**Methods: Blinding**	11a	If done, who was blinded after assignment to interventions and how.	4
	11b	If relevant, description of the similarity of interventions.	Not applicable; participants received rehabilitation with or without adjunctive FMS.
**Methods: Statistical methods**	12a	Statistical methods used to compare groups for primary and secondary outcomes.	9-10
	12b	Methods for additional analyses, such as subgroup analyses and adjusted analyses.	9-10
**Results: Participant flow**	13a	For each group, the numbers of participants who were randomly assigned, received intended treatment, and were analyzed for the primary outcome.	10-11; Figure 2
	13b	For each group, losses and exclusions after randomization, together with reasons.	10-11; Figure 2
**Results: Recruitment**	14a	Dates defining the periods of recruitment and follow-up.	10
	14b	Why the trial ended or was stopped.	10
**Results: Baseline data**	15	A table showing baseline demographic and clinical characteristics for each group.	11-12; Table 1
**Results: Numbers analyzed**	16	For each group, number of participants included in each analysis and whether the analysis was by original assigned groups.	10-11; Figure 2
**Results: Outcomes and estimation**	17a	For each primary and secondary outcome, results for each group and estimated effect size and its precision.	12-14; Table 2
	17b	For binary outcomes, presentation of both absolute and relative effect sizes is recommended.	Not applicable as a primary binary outcome; Fredericson grade distributions are reported descriptively.
**Results: Ancillary analyses**	18	Results of any other analyses performed, including subgroup analyses and adjusted analyses, distinguishing pre-specified from exploratory.	Post-hoc reliability analysis of BME extent measurements was performed and is reported in Section 3.3. 14
**Results: Harms**	19	All important harms or unintended effects in each group.	10
**Discussion: Limitations**	20	Trial limitations, addressing sources of potential bias, imprecision, and, if relevant, multiplicity of analyses.	16-17
**Discussion: Generalisability**	21	Generalisability/external validity of the trial findings.	16-17
**Discussion: Interpretation**	22	Interpretation consistent with results, balancing benefits and harms, and considering other relevant evidence.	14-17
**Other information: Registration**	23	Registration number and name of trial registry.	4; 18
**Other information: Protocol**	24	Where the full trial protocol can be accessed, if available.	18

Section/Topic	Item No.	Checklist item	Reported on page No.
**Other information: Funding**	25	Sources of funding and other support, and role of funders.	18

Note: Page numbers correspond to the manuscript version prepared for submission. Items marked as not applicable are not expected to be reported as substantive trial procedures.
