# Supplementary material for: Effects of Functional Magnetic Stimulation on Pain, Function, and MRI-Derived Outcomes in Athletes with Tibial Bone Stress Injury: A Randomized Controlled Trial
Source: J Funct Morphol Kinesiol. 2026 Aug 14;11(3):317. doi: 10.3390/jfmk11030317 (PMC13511723; doi:10.3390/jfmk11030317)
Supplement: Supplementary file 1 [file jfmk-11-00317-s001.zip › Supplementary Material.pdf]

## Supplementary Material

## Table S1. Standardized Physiotherapy Rehabilitation Program

| Phase | Goals | Exercise Category | Exercise | Load / Intensity | Volume | Progression Criteria |
|---|---|---|---|---|---|---|
| Week 1 | Pain reduction, controlled loading | Isometric loading | Bilateral calf raise holds | Bodyweight → +10–20% BW, RPE 6–7 | 5 × 30–45 sec | Pain ≤3/10, no next-day increase |
| | | Strength (low-load) | Leg press (bilateral) | 50–60% 1RM | 3 × 12 | Controlled execution |
| | | Mobility | Ankle dorsiflexion, calf stretching | Bodyweight | 3 × 10–12 / 30 sec | Pain-free ROM |
| | | Neuromuscular control | Single-leg stance | Bodyweight | 3 × 30 sec | Stable control |
| | | Locomotion | Walking | Symptom-guided | 20–30 min | Pain-controlled exposure |
| Week 2 | Progressive reloading | Heavy slow resistance | Single-leg calf raises | +20–40% BW, RPE 7–8 | 4 × 8–10 | Pain ≤3/10 |
| | | Strength | Split squats | Moderate load, RPE 7 | 3 × 8–10 | Movement quality |
| | | Strength | Romanian deadlift | Moderate load | 3 × 8 | Controlled tempo |
| | | Load transfer | Step-downs | Bodyweight → light load | 3 × 10 | No symptom increase |
| | | Locomotion | Fast walking | Symptom-guided | 30–40 min | Increased tolerance |
| Week 3 | Dynamic loading, energy storage | Strength | Single-leg calf raises | +40–60% BW, RPE 8 | 4 × 6–8 | High tolerance |
| | | Eccentric loading | Heel drops | Bodyweight → load | 3 × 10 | Controlled eccentric |
| | | Plyometrics (low-level) | Double-leg pogo jumps | Low amplitude | 3 × 12 | Pain-free execution |
| | | Dynamic drills | Skipping drills | Submaximal | 3 × 20 m | No symptom increase |
| | | Running exposure | Walk–run intervals | 1:2 ratio | 10–15 min | Symptom stability |
| Week 4 | Return-to-impact loading | Strength (high-load) | Single-leg calf raises | 60–80% BW equivalent, RPE 8–9 | 4 × 6 | High load tolerance |
| | | Plyometrics | Single-leg hops | Controlled amplitude | 3 × 8 | Pain-free |
| | | Plyometrics | Forward bounds | Moderate intensity | 3 × 6 | Controlled landing |
| | | Running progression | Run–walk intervals | 2:1 ratio | 15–20 min | No symptom exacerbation |
| | | Sport-specific | Change of direction drills | Submaximal | 3 × 20 m | Functional readiness |

Participants progressed through the rehabilitation program according to a symptom-guided loading model. Exercise intensity and volume were increased only when pain during and after activity remained ≤3/10 and no exacerbation of symptoms was reported on the following day. The program was based on contemporary principles of graded mechanical loading, progressive return to running, and management of tibial load tolerance in bone stress injury.

The presented exercise progression reflects a structured representation of the rehabilitation framework applied in the study. In clinical practice, exercise selection and loading parameters were individualized according to symptom response; however, progression consistently followed the symptom-guided model described above.
